# Mechanical Sensitivity Improvement of CL-20 by Using Crystal Passivation: A Brief Review

**DOI:** 10.1021/acsomega.4c11210

**Published:** 2025-06-25

**Authors:** Ruixiao Li, Weiqiang Pang, Yang Zhang, Djalal Trache, Luigi T. DeLuca

**Affiliations:** † National Key Laboratory of Energetic Materials, 58300 Xi'an Modern Chemistry Research Institute, Xi'an 710065 China; ‡ Energetic Materials Laboratory (EMLab), Teaching and Research Unit of Energetic Processes, Ecole Militaire Polytechnique, BP 17, Bordj El-Bahri, 16046 Algiers, Algeria; § Space Propulsion Laboratory (SPLab), Department of Aerospace Science and Technology, Politecnico di Milano, 20156 Milan , Italy

## Abstract

Energetic materials (EMs) have important application value in the military and civil fields. But at the same time, EMs will burn or explode when they are impacted or rubbed. 2,4,6,8,10,12-Hexanitro-2,4,6,8,10,12-hexaazaisowurtzitane (CL-20) is one of the typical energetic compounds with high energy, whose density is 2.04 g cm^–3^ and velocity of detonation is 9380 m s^–1^. However, its high mechanical sensitivity (impact sensitivity and friction sensitivity) has become one of the major problems that limit its application in solid propellants and explosives. Consequently, the mitigation of CL-20's mechanical sensitivity has remained a critical research objective in the field of EMs. In this review, the passivation of CL-20, including crystal control, cocrystallization, surface coating, spheronization, and nanonization, is summarized. Different methods significantly reduce the sensitivity of CL-20, with the lowest impact sensitivity reaching 35 J. In addition, research directions on the passivation of CL-20 in the future were proposed, promoting the application of CL-20 in energetic systems.

## Graphical abstract

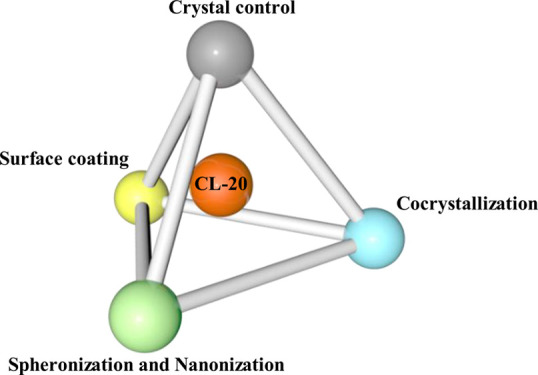

## 1 Introduction

Energetic materials (EMs) can release a large amount of energy in the process of chemical reaction, which has been widely used in many research fields. 2,4,6,8,10,12-Hexanitro-2,4,6,8,10,12-hexaazaisowurtzitane (CL-20) with a polycyclic cage multinitramine structure is one of the most energetic explosives, which was synthesized in the United States first in 1987.^1^ The physicochemical properties of several important high EMs are listed in Table 1.^2–6^ Compared with other EMs such as hexahydro-1,3,5-trinitro-1,3,5-triazine (RDX), octahydro-1,3,5,7- tetranitro-1,3,5,7-tetrazocine (HMX), and 1,1-diamino-2,2-dinitroethylene (FOX-7), it was considered as a potential EM with high density (ρ = 2040 kg m^–3^), a positive heat of formation (Δ*H*_f_ = 454 kJ mol^–1^), high detonation velocity (9380 m s^–1^), and preferable oxygen balance (−11.0) and detonation pressure (42 MPa).^2^

**Table 1:** Physicochemical Properties of Several Important EMs^a^

sample	Δ*H*_f_/(kJ mol^–1^)	OB%	ρ/(kg m^–3^)	*D*/(km s^–1^)
CL-20	+454	–11.0	2040	9.38
HMX	+76	–22.0	1910	9.10
RDX	+63	–22.0	1820	8.75
ADN	–135	+26.0	1808	6.30
AP	–298	+35.0	1950	/
TNAZ	+26.8	–16.7	1860	8.80
TKX-50	+446.6	–27.1	1877	9.70
FOX-7	+575	–21.6	1894	8.87

a Note: Δ*H*_f_, heat of formation, kJ mol^–1^; OB, oxygen balance; ρ, density, kg m^–3^; *D,* velocity of detonation, km s^–1^.

At the same time, EMs are susceptible to explosion, combustion, or decomposition when they are subjected to impact or friction, which brings great challenges to their application. The mechanical sensitivity of EMs, including impact and friction sensitivities, is an important parameter to characterize their safety performance. Impact sensitivity refers to the degree of difficulty of explosion or combustion of materials under an external mechanical impact, which is represented by the critical impact energy corresponding to its 50% explosion probability.^7^ Friction sensitivity reflects the difficulty of combustion or explosion of materials under mechanical friction, which is usually the critical friction corresponding to the 50% explosion probability or the percentage of explosion or combustion under a certain friction. These two parameters are closely related to the crystal form, microstructure, particle size, and morphology of EMs. For CL-20, extremely high mechanical sensitivity (impact sensitivity is 2.00–4.40 J, solvent/antisolvent method) has become one of the important factors limiting its application compared with RDX (impact sensitivity is 7.50 J) and HMX (impact sensitivity is 7.50 J).

In order to reduce the mechanical sensitivity of CL-20, many efforts have been made by researchers worldwide.^8–12^ Experimental investigations have revealed that ε-CL-20 exhibits lower impact sensitivity compared to other forms, with a measured impact sensitivity threshold of 13.13 J.^13,14^ This value significantly exceeds those of other forms, 10.14 J for α-CL-20, 11.86 J for β-CL-20, and 12.20 J for γ-CL-20. Therefore, controlling the crystal form of CL-20 to ε*-*CL*-*20 has become a highly concerning direction for researchers. Cocrystallization of CL-20 with other compounds represents an effective strategy to address its high sensitivity. However, discrepancies exist in current research regarding CL-20 cocrystals, with some scholars suggesting they may constitute a form of composite material. To facilitate classification, this review defines cocrystals as systems in which molecules achieve a mutually stabilized structure through noncovalent interaction, without exhibiting distinct phase separation at the nanoscale.^10,15^ For instance, when the CL-20/1-methyl-3,4,5-trinitropyrazole cocrystal was obtained in ethyl acetate, the impact sensitivity of the cocrystal was 28.07 J.^16^ Compared with raw CL-20 (6.37 J), the sensitivity of the cocrystal decreased significantly. Furthermore, surface coating is an effective method to reduce the sensitivity of EMs under external stimulations. For example, nitrocellulose (NC) and polyazide glycidyl ether (GAP) were used as the composite coating agent for CL-20. When the mass ratio of GAP/NC@CL-20 was 2:3:95, the impact sensitivity of the sample decreased to 9.00 J, which decreased the mechanical sensitivity of CL-20 significantly.^17^ In addition, the spheronization and nanonization of CL-20 could also change its mechanical sensitivity by changing the energy transfer between the crystal grains. One example is that near-spherical CL-20 (*D*_50_, average particle size is 685.4 nm) was prepared by a supercritical fluid solution, and the impact sensitivity decreased by 4.54 J compared with microsized raw CL-20.^18^

In this review, a discussion on passivation of CL-20 crystal transformation is presented, including crystal control, cocrystallization, surface coating, spheronization, and nanonization from the viewpoint of numerical simulation and experiment aspects (Figure 1). Based on the survey and comparison, the effects of passivation are summarized, its advantages and disadvantages are compared, and future development prospects are forecast.

**Figure 1:**
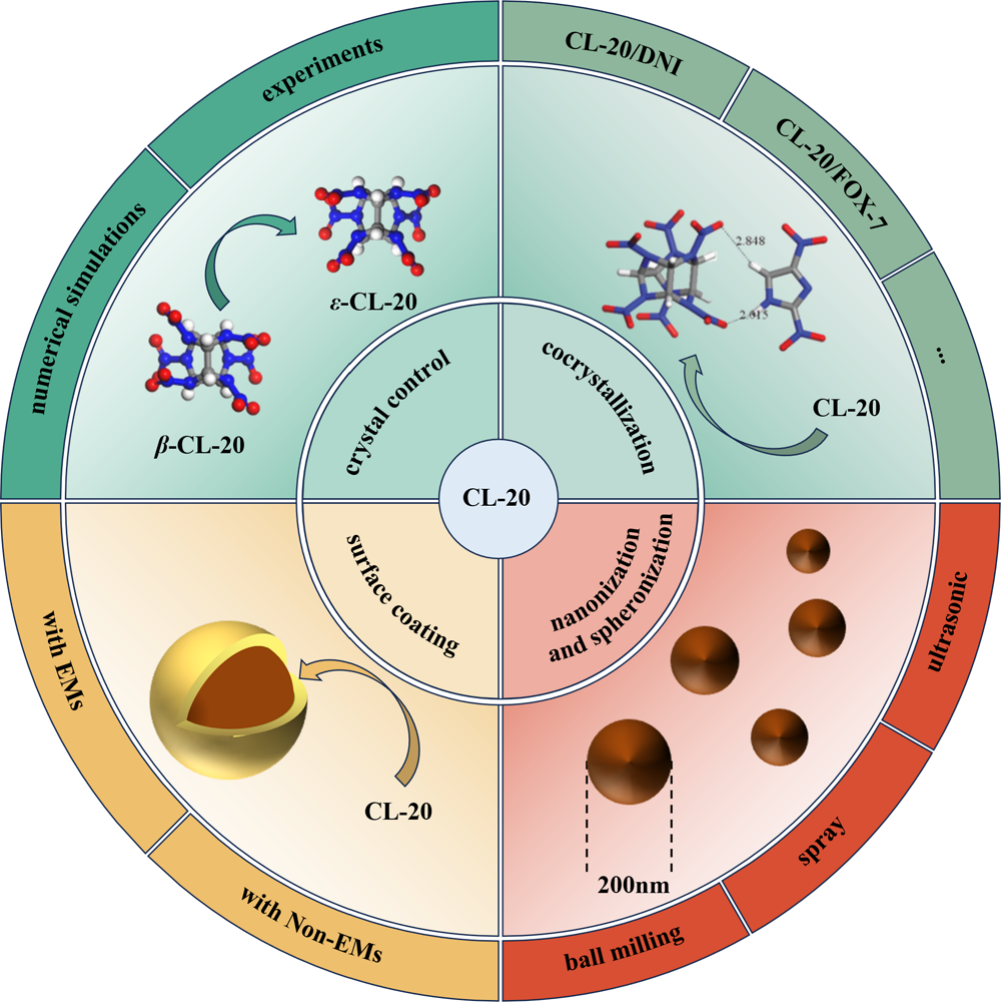
Different methods for the passivation of CL-20.

## 2 Crystal Control

### 2.1 Numerical Simulations

Previous studies have shown that the ε- crystal form is the least sensitive in four crystal forms of CL-20.^13,14^ Therefore, researchers always attempt to prepare ε-CL-20 as purely as possible. The polymorphic transformation between ε-CL-20 and β-CL-20 has been simulated by short-range order and orientational order parameters.^19^ It is found that the peaks of the two crystal forms are close at 227 °C, suggesting that the temperature of crystal transformation is at about 227 °C. This is consistent with the results obtained in previous experiments.^20^ In addition, linear analysis indicates that the surface electrostatic potentials affect transformation, which suggests that the process of polymorphic transformation can be affected by the polarity of the solvent. For instance, in the process of predicting the growth of the crystal, the general spiral growth model is more suitable for the polyhedral morphology growth of ε-CL-20. However, when the edge energy decreases (replacing ethyl acetate with methanol as a solvent), the spiral growth model is not applicable.^21^ Moreover, first-principles calculations and molecular dynamics (MD) simulations also confirm this viewpoint, and the transformational energy barriers (Δ*E*_s_) of α-, γ-, ε-, and β-crystal forms in different solvents and gaseous states are performed (Figure 2a).^22^ It can be seen that the solvent reduces Δ*E*_s_, and the difference in energy between these conformations is small, which indicates that CL-20 molecules are prone to conformational changes in solution and different molecular conformations of CL-20 can coexist in solution. Coherent energy density (CED) is also analyzed (Figure 2b). The CED of β-, γ-, anhydrous α-, and ε-CL-20 increased sequentially, which suggests ε-CL-20 is the most stable crystal thermodynamically.

**Figure 2:**
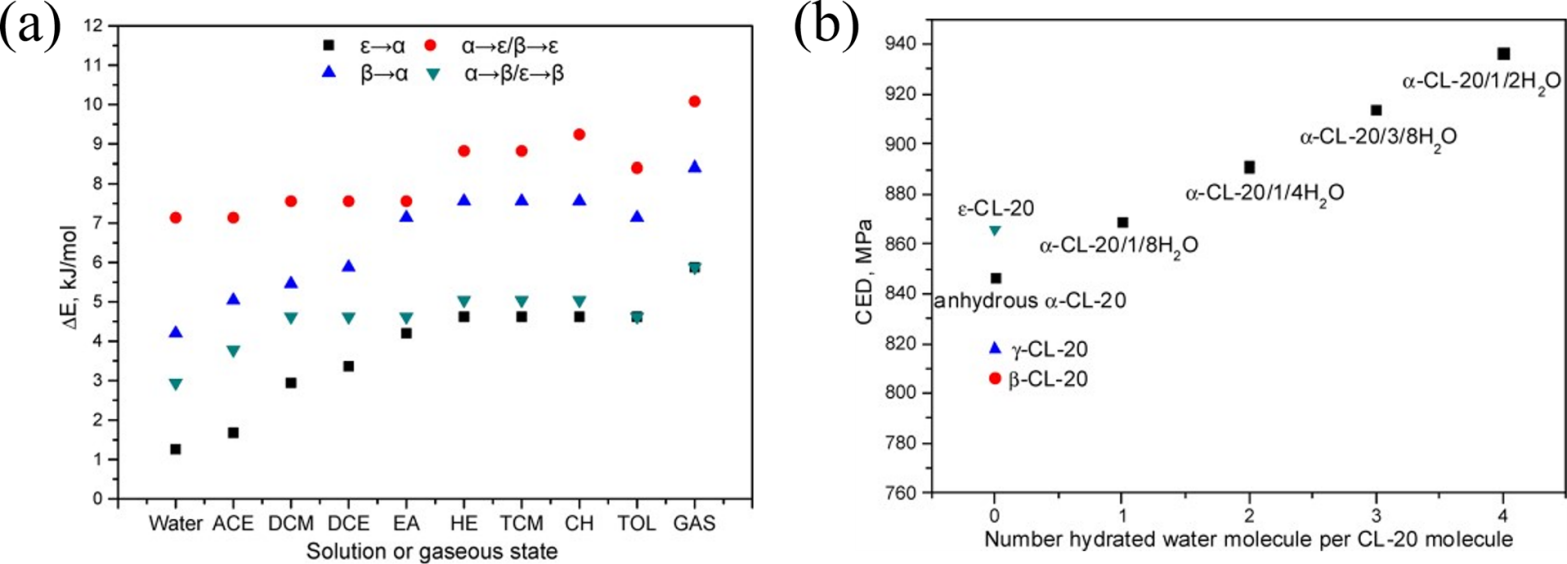
(a) Transformational energy barriers (Δ*E*_s_) of three CL-20 conformations in different solvents and gaseous state (ACE (acetone), DCM (dichloromethane), DCE (dichloroethane), EA (ethyl acetate), HE (heptane), TCM (carbon tetrachloride), CH (cyclohexane), TOL (toluene), GAS (gaseous state)); (b) CED of different CL-20 crystal forms (reproduced from ref 22 with permission).

### 2.2 Experiments

The influence of the solution on crystal transformation has also been explored in experiments. Due to the great thermodynamic driving force and fast nucleation rate, an approach was proposed to purify ε-CL-20 from β-CL-20 through solution-mediated polymorphic transformation (SMPT) in an ethyl acetate–chloroform mixed solvent.^23^ When the chloroform content is decreased, the transformation speeds up, providing a high solubility in solution. In addition, temperature is another important influencing factor. With the increase in temperature, the kinetic substitution of thermodynamics dominates, and the Avrami Erofeev model parameters are shown in Table 2. The values of *K* increase with increasing temperature, which suggests that the rate of crystal transformation can be accelerated by increasing the temperature. The effect of different additives on the crystal transformation of ε-CL-20 was investigated.^24,25^ Studies have shown that additives can modify the crystal morphology by adsorbing on the crystal surface, changing the surface free energy, and preventing the growth of some crystal planes.^26^ Different additives make the crystals grow into different shapes. Aminoacetic acid makes the crystal grow into a nearly spherical shape, and its impact sensitivity is the lowest, which is 25.77 J. For the rate of phase transition, the XRD analysis shows that the effect of additives on the crystal transformation from ε*-* to γ*-* crystal form can be divided into three types.^27^ The first type delays the transition process, and the transition rate is the same as that of neat ε-CL-20 after reaching the transition temperature. The second type delays the transition process as well, but once the transformation begins, the speed improves significantly. The third type accelerates the entire process of transformation.

**Table 2:** Kinetic Parameters of the SMPT Process from the β*-* to ε- Form at Different Temperatures (Reproduced from Ref 23 with Permission)

*T*/K	*K*	*N*	*R* ^2^
298.15	0.0060	0.2055	0.9979
303.15	0.0073	0.2264	0.9986
308.15	0.0091	0.2571	0.9989
318.15	0.0125	0.2656	0.9993

Defect nucleation has much influence on the crystal transformation of CL-20.^28^ For instance, the ε → γ isothermal phase of CL-20 was studied by situ diffuse reflectance infrared spectroscopy, which showed that when the degree of crystal transformation is between 1 and 18%, the apparent activation energy (*E*_a_) and the pre-exponential factor ln (A/s^–1^) are 150.6 and 38.1 kJ mol^–1^, respectively. When the degree of phase transition is between 18 and 94%, the *E*_a_ and pre-exponential factor ln (A/s^–1^) are 289.4 and 74.7 kJ mol^–1^, respectively. It is believed that the differences were caused by the defect nucleation mechanism in the early stage of crystal transformation.

In addition, it must be pointed out that 2.00–4.40 J is generally considered the impact sensitivity of CL-20 prepared by the solvent/antisolvent method. However, the phenomenon can be attributed to the formation of microbubbles or cracks resulting from rapid cooling or phase separation.^29^ The presence of microstructural defects (microbubbles and cracks) compromises the structural integrity of CL-20 crystals and exacerbates their mechanical sensitivity. Studies have shown that CL-20 with lower sensitivity can be obtained.^30^ For instance, the impact sensitivity of ε-CL-20 is 8.35 J, which is obtained through the recrystallization method.^31^ In addition, a green mechanical demulsification method under a strong fluid shearing force and intense high-frequency mechanical effects was proposed to process ε-CL-20.^32^ It can be found that the final product maintains the ε- crystal form, whose impact sensitivity decreased from 3.82 to 8.45 J. Scholars from the Beijing Institute of Technology carried out extensive characterization and research on different crystal forms of CL-20 in the early stage.^33–37^ Through a synthesis process involving the initial preparation of α-CL-20 followed by its conversion to ε-CL-20, they obtained ε-CL-20 exhibiting an impact sensitivity of 13.13 J.^14^

### 2.3 Summary

The different crystal forms of CL-20 have different properties. Previous studies have shown that the ε- form possesses lower mechanical sensitivity. Therefore, the current research mainly focuses on the transformation of other forms to the ε- form.^14^ The mechanism of crystal transformation is explored from thermodynamics or kinetics by MD simulations and first-principles calculations. Conditions during crystallization are the main ways to control a crystal, such as the selection of temperature and solvent.^38^ In recent years, the influence of other substances (such as additives and crystal growth modifiers) in the system on the crystal structure of products has also been studied, which indicates that related research has been further enriched over time. In addition, researchers have prepared crystals with fewer defects by recrystallization and other methods. The obtained crystals have a lower impact sensitivity than the crystals obtained by the traditional solvent–antisolvent method.

## 3 Cocrystallizations

### 3.1 CL-20/DNI

Cocrystal passivation technology can realize the noncovalent bond between high-energy sensitive molecules and insensitive molecules at the molecular level.^10,39^ The sensitive CL-20 and insensitive crystals form multicomponent molecular crystals by intermolecular forces, which can improve their stability and reduce their sensitivity.^40^ For example, the number of “entrance mode” phonons is correlated with the impact sensitivity linearly and positively.^41,42^ The first principle is used to study the sensitivity of the CL-20/1,4-DNI cocrystal.^43^ The density of phonon states was calculated, and the “entrance mode” phonon numbers of ε-CL-20, ε-CL-20/1,4-DNI, and 1,4-DNI were 44, 26, and 13, respectively. These results indicate that the order of impact sensitivity is [ε-CL-20] > [CL-20/1,4-DNI] > [1,4-DNI]. Furthermore, the CL-20/1,4-DNI cocrystal in a molar ratio of 1:1 can also be obtained by the evaporation solvent method in ethyl acetate.^44^ The impact sensitivities of the CL-20/1,4-DNI cocrystal, ε-CL-20, and 1,4-DNI are 10, 2.5, and 14 J, respectively, showing that the sensitivity of the cocrystal is greatly reduced compared with raw ε-CL-20. Further research indicates that the formation of CL-20/1,4-DNI cocrystals mainly depends on three main types of intermolecular forces (Figure 3). These forces make the cocrystal more stable and less sensitive undoubtedly.^45–47^

**Figure 3:**
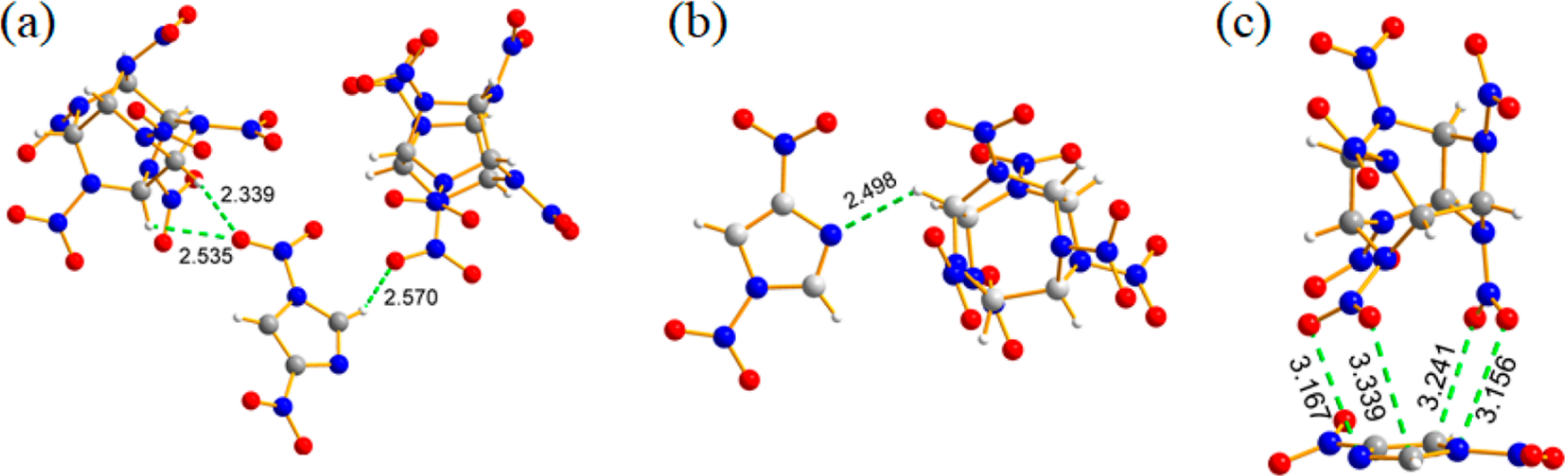
Intermolecular interactions and Hirshfeld surface of the CL-20/1,4-DNI cocrystal: (a) CH···O hydrogen-bonding interactions; (b) CH···N hydrogen-bonding interactions; and (c) NO_2_–π interactions (reproduced from ref 44 with permission).

In addition, a spray-drying device was used to prepare the CL-20/2,4-DNI cocrystal with 97.65% yield.^48^ The *D*_50_ of the cocrystal samples was 518.25 nm. The sensitivity test results of the samples show that the impact sensitivity of the CL-20/2,4-DNI cocrystal decreased from 9.18 to 29.60 J, and the friction sensitivity decreased from 120 to >360 N, compared with raw CL-20. It was found that the sensitivity of the cocrystal is significantly lower than the raw CL-20, which implies that there are other interacting forces in the cocrystal. Further theoretical calculations show that this force is a CH···O bond and exists in four configurations.

### 3.2 CL-20/FOX-7

The CL-20/FOX-7 (molar rate is 2:1) cocrystal was prepared by a spray flash evaporation method.^49^ Compared with the impact sensitivity of CL-20 (5.88 J), the impact sensitivity of the CL-20/FOX-7 cocrystal is increased to 13.13 J, attributed to the closer hydrogen-bond combination between different molecules. Analyses on the binding energy of the CL-20/FOX-7 cocrystal in molar ratios of 10:1, 9:1, 8:1, 7:1, 6:1, 5:1, 4:1, 3:1, 2:1, and 1:1 show that the sensitivity of the sample is lower with a decrease in the molar ratio. In addition, the binding energy is the highest when the molecular molar ratio is 1:1.^50^

### 3.3 CL-20/HMX

With the existence of a high-boiling-point antisolvent, a cocrystal can be obtained by evaporation of a solution in a saturated solvent.^51^ In the experiment, β-HMX and ε-CL-20 were used as raw materials, anhydrous acetone was used as a solvent, and xylene was selected as the nonsolvent. Results showed that HMX in the prepared CL-20/HMX cocrystal whose molar ratio is 2:1 was the β*-* crystal form, and CL-20 was a mixture of β*-* and γ*-* crystal forms. The final sensitivity test data are shown in Table 3. The results show that the sensitivity of the CL-20/HMX cocrystal is reduced compared with the raw CL-20. The same conclusion is obtained from the test results of the CL-20/HMX cocrystal prepared by other methods.^52^ Moreover, when the molar ratio is also 2:1, the impact sensitivity of the CL-20/HMX cocrystal prepared by the microchannel method reaches 18 J and the friction sensitivity reaches 96 N.^53,54^ This may be due to the fact that the morphology of the obtained crystal is flower clusters and platelets, and the thickness of platelets is 200–600 nm (Figure 4).

**Table 3:** Results of Impact and Friction Sensitivity Analysis (Reproduced from Ref 51 with Permission)

sample	impact insensitivity/J	friction insensitivity/N
raw ε-CL-20	4.90–5.49	84–90
raw β-HMX	8.82–9.80	190–200
CL-20/HMX cocrystal	8.43–9.41	300–330

**Figure 4:**
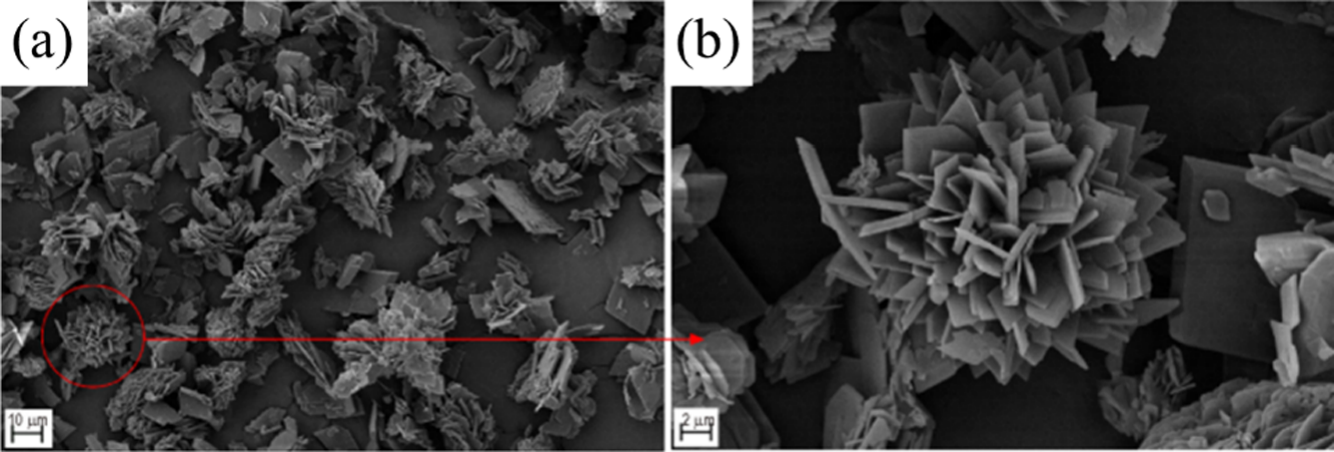
SEM images of the CL-20/HMX cocrystal at different scales. (a) Scale bar: 10 μm; (b) scale bar: 2 μm (reproduced from ref 53 with permission).

There are many MD studies on the CL-20/HMX cocrystal, which not only indicate that the CL-20/HMX cocrystal is less sensitive than CL-20 ^55^ but also point out that crystal defects enhance the sensitivity of the cocrystal.^56^ The influence of external electric fields on cocrystal sensitivity has also been discussed.^57^ In addition, the influence of molecular molar ratio on sensitivity has been studied.^58^ ε-CL-20 and β-HMX were selected to simulate cocrystals according to molecular molar ratios of 10:1, 9:1, 8:1, 7:1, 6:1, 5:1, 4:1, 3:1, 2:1, and 1:1. Analysis on binding energies of different samples shows that the binding energy gradually increases with a decrease of the molar ratio. In all samples, the sensitivity of the sample is the lowest when the molar ratio is 1:1.

### 3.4 CL-20/TNT

The CL-20/TNT cocrystal in a molar ratio of 1:1 was prepared by a spray-drying method.^59^ The SEM of the samples (Figure 5) shows that the particle size distribution of the raw material CL-20 is 30∼300 μm, and the particle size of the CL-20/TNT cocrystal is less than 1 μm. The small grain diameter causes the sensitivity of the sample to decrease as well. The results indicate the cocrystal’s impact sensitivity is 12.08 J, while the raw CL-20's impact sensitivity is 3.21 J. The average particle size of the prepared cocrystal grain size is about 1 mm using another method.^60^ According to the test experiments, the cocrystal’s impact sensitivity was 10.54 J, which increased by 7.35 J compared with raw CL-20. In addition, explosion probability caused by friction is reduced by 51% (pendulum weight, 1.50 ± 0.01 kg; sample mass, 20 ± 1 mg; relative pressure, 4.9 MPa; and swaying angle, 90°).

**Figure 5:**
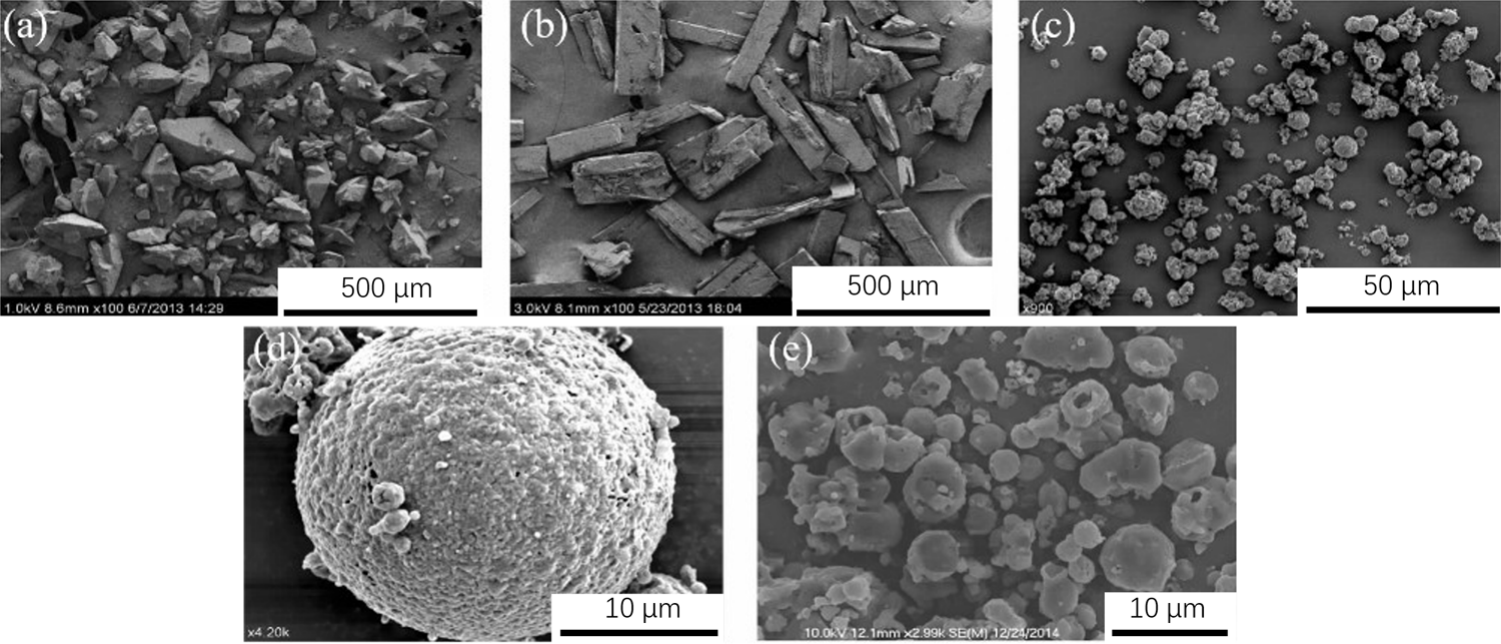
SEM photos of explosive samples. (a) Raw CL-20 (×100); (b) raw TNT (×100); (c) CL-20/TNT cocrystal samples (×900); (d) CL-20/TNT cocrystal samples (×4200); and (e) spray-drying CL-20 (×2990) (reproduced from ref 59 with permission).

Furthermore, based on the bond interaction energies and cohesion energy densities obtained from MD simulations, it can be inferred that CL-20/TNT cocrystals are more stable and less sensitive compared to raw CL-20.^61^

### 3.5 Other Binary Cocrystallizations

In addition, studies on other binary cocrystals have shown that CL-20/2,4-dinitro-2,4-diazapentane (DNDAP), CL-20/dinitrobenzene (DNB), CL-20/trans-1,4,5,8-tetranitro-1,4,5,8-tetraazadecalin (TNAD), CL-20/3-nitro-1,2,4-triazol-5-one (NTO), CL-20/1-amino-3-methyl-1,2,3-triazoliumnitrate, and CL-20/2,4,6-trinitro-3-bromoanisole have better stability than raw CL-20 at different angles.^62–68^

Meanwhile, the CL-20/7H-trifurazano [3,4-b:3′,4’-f:3”,4”-d]­azepine (TFAZ) cocrystal in the molar ratio of 1:1 prepared by the self-assembly method (*D*_50_ of 8.57 μm) has a smaller particle size than that prepared by a slow solvent evaporation method (*D*_50_ of 1 mm), whose impact sensitivity is 8.232 J and the explosion probability (*P*) toward friction is 38% (pendulum angle of 66°, sample mass of 20 mg, and relative pressure of 2.45 MPa).^69^ In addition, the solvent/nonsolvent method was used to prepare the CL-20/dihydroxylammonium-5,5′-bistetrazole-1,1’-diolate (TKX-50) cocrystal. In comparison to raw CL-20, which exhibits an impact sensitivity of 3.92 J with a particle size of approximately 1 μm, the CL-20/TKX-50 cocrystal demonstrates a significantly reduced impact sensitivity of 16.66 J, despite having a larger particle size of around 10 μm.^70^ The minimum explosion energy of the CL-20/1,3,5-triamino-2,4,6-trinitrobenzene (TATB) cocrystal sample with an average grain size of 3∼5 μm is 3 J.^71^ Except for these, the sensitivity of CL-20/DNDAP, CL-20/dinitrotoluene, and CL-20/nitroguanidine (NQ) cocrystals also decreased, compared with raw CL-20.^72–74^

### 3.6 Summary

Although changing the crystal form partly reduces the sensitivity of CL-20, its effect is limited. Researchers must further search for methods to reduce the sensitivity. One method is to combine it with other substances with lower sensitivity to form a cocrystal. At present, research on the cocrystal of CL-20 mainly includes simulations and experiments. In terms of simulation, researchers employed MD simulations to generate data, including an inlet mode, trigger bond length, and binding energy, to characterize the molecular interactions. Subsequently, these parameters were utilized to deduce the mechanical sensitivity trends of the sample. Experiments show that the impact sensitivity of the CL-20/2,4-DNI cocrystal is the lowest, and it is significantly higher than that of the CL-20/1,4-DNI cocrystal (Figure 6). This may be due to the existence of more different intermolecular forces, which make the overall structure more stable. At the same time, it can be seen that the impact sensitivity of the samples prepared by different methods of the same material is also different, which may be due to the different binding degrees of the two molecules in the materials. Besides these, there are also some unsatisfactory results, such as the CL-20/TATB cocrystal. The ratio of raw materials and the preparation method may lead to this result, which is worthy of further study by researchers. Similarly, the cocrystal also has different degrees of improvement in friction sensitivity (Table 4).

**Figure 6:**
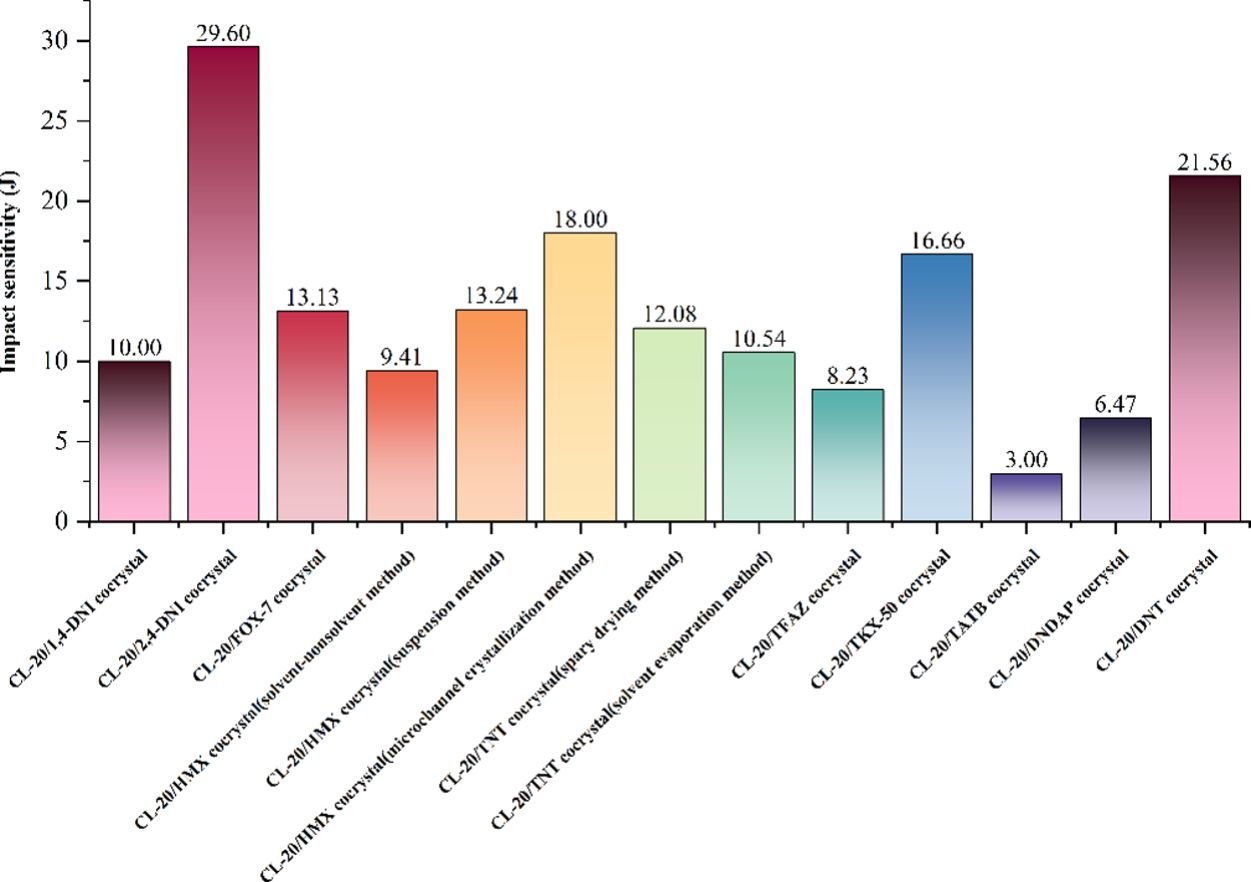
Passivation effect of the cocrystal on the impact sensitivity of CL-20.

**Table 4:** Passivation Effect of the Cocrystal on the Friction Sensitivity of CL-20^a^

sample	passivation effects	ref.
CL-20/2,4-DNI cocrystal	FS decreased from 120 to >360 N	^48^
CL-20/HMX cocrystal (solvent–nonsolvent method)	FS decreased to 96 N	^52^
CL-20/TNT cocrystal (solvent evaporation method)	FS decreased from 100 to 49%	^60^
CL-20/TFAZ cocrystal	FS decreased to 38%	^69^
CL-20/DNDAP cocrystal	FS decreased from 100 to 48%	^72^
CL-20/NQ cocrystal	FS decreased from 100 to 52%	^74^

a Note: FS is friction sensitivity.

## 4 Surface Coating

### 4.1 Surface Coating with EMs

Surface coating, with the advantages of mature technology, simple method, and low cost, is one of the most commonly used techniques to reduce the sensitivity of EMs.^75^ Crystal sensitivity can be improved by surface coating through enhancing lubrication between grains, reducing external stimulation to crystals (Figure 7).

**Figure 7:**
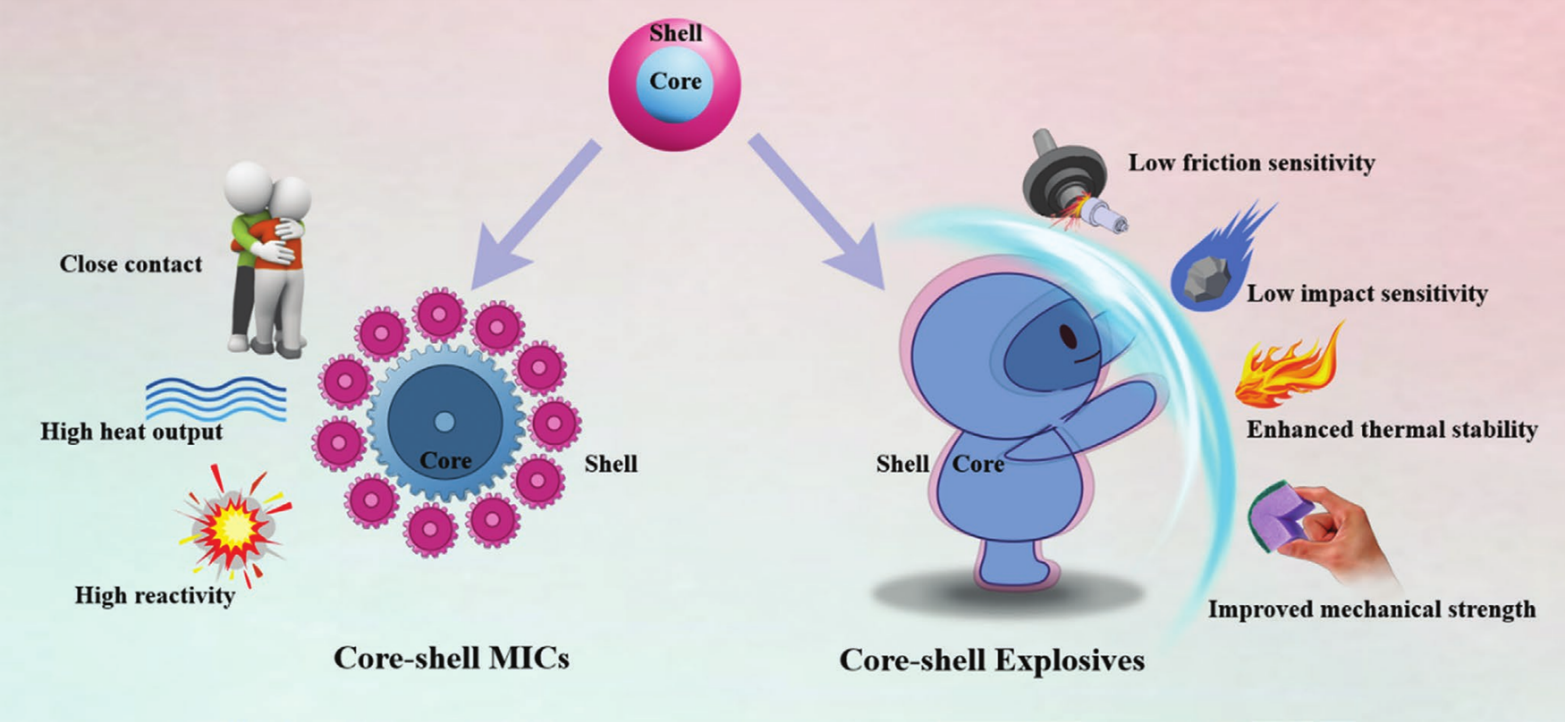
Effect of core–shell structure-coated EMs on their properties (reproduced from ref 76 with permission).

In order to balance the energy density and the sensitivity of CL-20, a feasible method is to coat it with EMs. For instance, TNT was introduced to coat CL-20, and the CL-20@TNT composite in a molar molecular ratio of 45:55 was proposed by a spray-drying method.^77^ The average particle size of the prepared CL-20@TNT composite is 30∼50 μm, which is lower than that of raw CL-20 (57.16 μm). The impact sensitivity of the prepared composite is 17.49 J, which is much higher than that of raw CL-20 (6.35 J). The emulsification method was used to prepare the CL-20@2,6-diamino-3,5-dinitropyrazine-1-oxide (LLM-105) composite.^78^ It can be seen that there is a decrease in sensitivity after being coated by LLM-105 (Figure 8). Similarly, the sensitivity of CL-20 also significantly decreases after being coated by TATB.^79^

**Figure 8:**
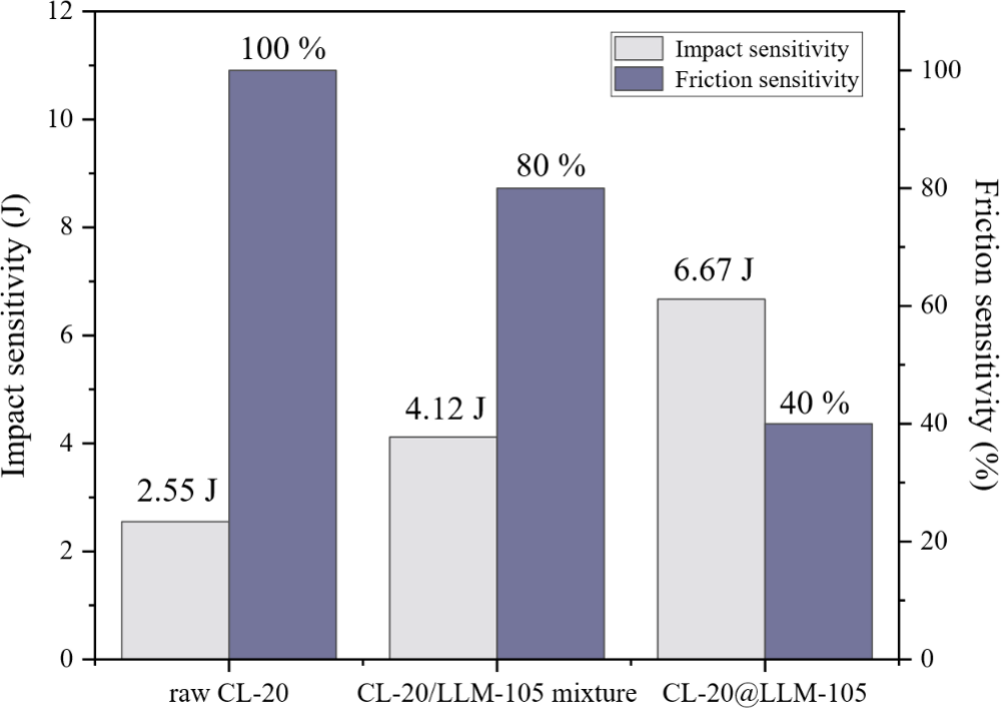
Impact sensitivity and friction sensitivity of CL-20, CL-20/LLM-105 physical mixtures, and CL-20@LLM-105 composites. The friction sensitivity test conditions are a pendulum weight of 1.5 kg and a sample mass of 20 ± 1 mg.

Another instance is the metal–organic framework (MOF) as a kind of potential EM. CL-20@MOF-199 was prepared via the spray-drying self-assembly technique.^80^ The sensitivity test results show that as the content of MOF-199 increases, the sensitivity of the composites decreases. Because the MOF-199 shell structure can prevent external heat and shock from stimulating the internal CL-20, which reduces the probability of hot spot formation, mechanical sensitivity is decreased greatly.

### 4.2 Surface Coating with Non-EMs

Estane5703, wax, and stearic acid, as traditional lubricants, have appeared extensively in research on coating to reduce the sensitivity of CL-20.^81–85^ For example, wax and Estane5703 are used to coat CL-20.^86^ Compared with raw CL-20 (*D*_50_ of 40 μm), the impact and fraction sensitivity of CL-20/wax/Estane5703 with a mass ratio of 96:2:2 (*D*_50_ is 600∼900 μm) decreased by 60 and 52%, respectively (Table 5).

**Table 5:** Test Results of Sensitivity Performance of Different Samples (Reproduced from Ref 86 with Permission)^a^

sample	mass ratio	impact sensitivity/%	friction sensitivity/%
raw CL-20		100	100
CL-20@Estane5703	96:4	60	72
CL-20/@wax(internal)/Estane5703(external)	96:2:2	40	48
CL-20@wax(external)/Estane5703(internal)	96:2:2	76	72
CL-20@wax	96:4	68	60

a Note: The friction sensitivity test conditions are a pendulum angle of 90°, sample mass of 20 mg, and relative pressure of 3.92 MPa.

In addition, graphene foam (GF) was used to coat CL-20 to reduce its sensitivity.^87^ Research shows that when the mass ratio of CL-20 to GF is 98:2, the impact sensitivity decreased from 2.0 to 4.5 J and the friction sensitivity decreased from 108 N to about 252 N. In another instance, a core–shell–shell structural model was proposed for CL-20 coated by polydopamine (PDA) and graphene oxide (GO).^88^ Figure 9 shows the test results of the composites, and it is clear that GO has a desensitivity effect on CL-20. The reasons may be as follows: (1) GO effectively reduces the impact energy to CL-20 as a soft material; (2) GO has good lubricity, which can reduce the impact and friction by consuming energy; and (3) GO has high thermal conductivity, which prevents the formation of thermal stress concentration and hot spot to a certain extent under external stimulation. A similar effect occurs when other types of graphene or nitrofullerene (NC_60_) are selected to coat CL-20.^89–93^

**Figure 9:**
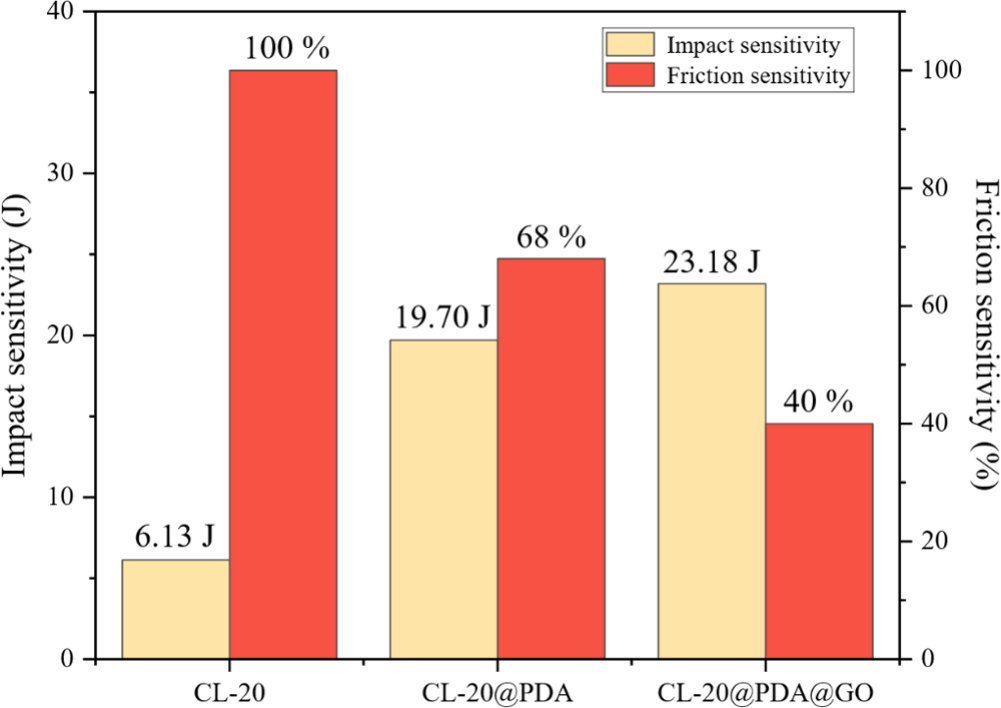
Histogram of impact sensitivity and friction sensitivity accumulation of CL-20, CL-20@PDA, and CL-20@PDA/GO. The friction sensitivity test conditions are a sample mass of 20 mg, pendulum of 1.5 kg, inclination of 80°, and gauge pressure of 2.45 MPa.

### 4.3 Summary

Surface coating is a common operation in chemical research and can also be applied to reduce the sensitivity of CL-20. Research on the surface coating of CL-20 includes using EMs or non-EMs as coating materials. The advantage of employing EMs as coating agents lies in their ability to preserve a high energy content while decreasing sensitivity, although the variety of EMs currently under investigation remains limited. Research on the application of non-EMs as coating materials is relatively extensive, with the majority of prior studies incorporating wax into their systems and exploring various composition ratios and preparation conditions. In the investigation of the influence of surface coating on impact sensitivity (Figure 10), while CL-20@M550/GO demonstrates the most favorable performance, the different outcomes of other GO-based coating studies indicate that, in addition to GO itself, the contributions of other components are also crucial. Most energetic coating materials not only show satisfactory results in maintaining energy output but also greatly reduce impact sensitivity. It is worth noting that surface coating significantly reduces friction sensitivity (Table 6), which should be attributed to the lubricating properties of the selected materials (wax or GO), effectively reducing interfacial friction in the composite system.

**Figure 10:**
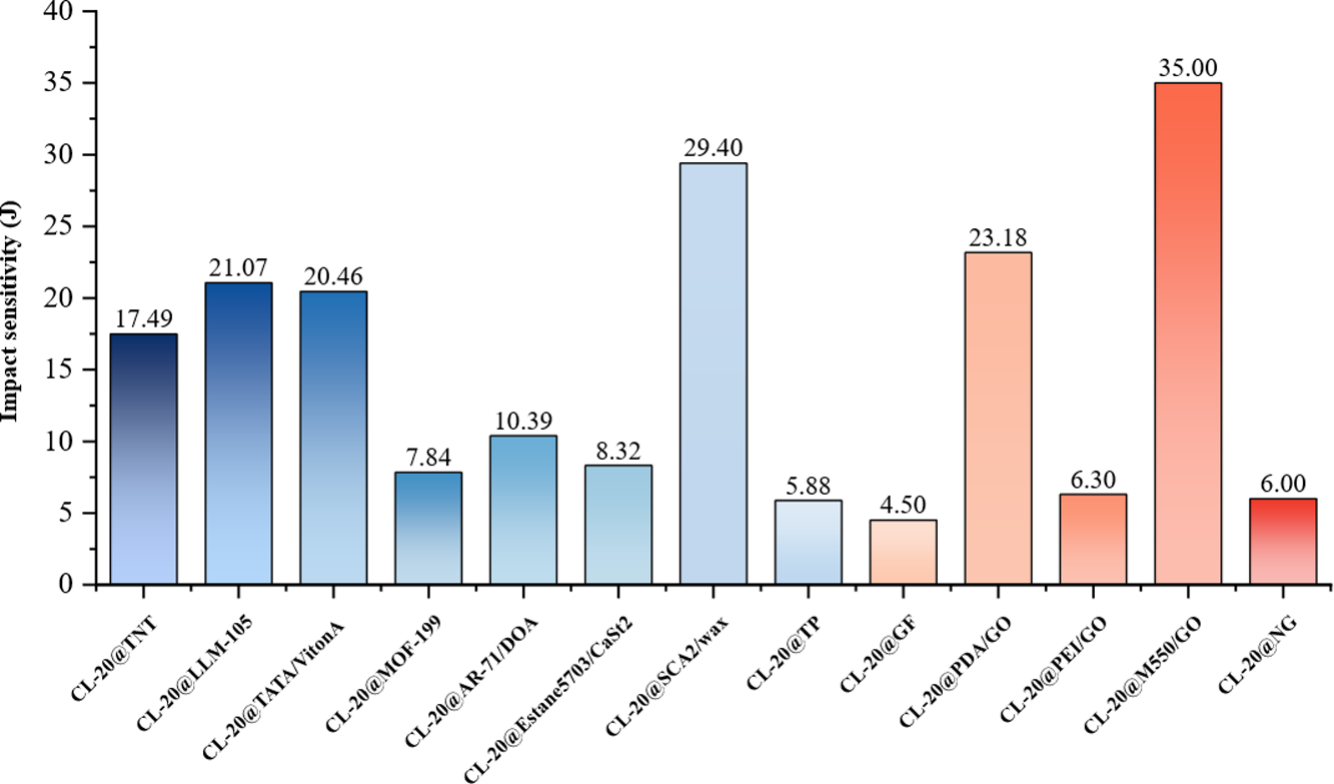
Passivation effect of surface coating on the impact sensitivity of CL-20.

**Table 6:** Passivation Effect of Surface Coating on the Friction Sensitivity of CL-20

samples	passivation effects	ref.
CL-20@LLM-105	FS decreased from 100 to 40%	^78^
CL-20@TATB/VitonA	FS decreased from 100% to about 40%	^79^
CL-20@MOF-199	FS decreased from 192 N to about 240 N	^80^
CL-20@Estane5703/CaSt_2_	FS decreased to 0%	^82^
CL-20@PW/SA	FS decreased from 100 to 30%	^83^
CL-20@SCA2/wax	FS decreased from 96 N to about 360 N	^84^
CL-20@wax/Estane5703	FS decreased from 100 to 48%	^86^
CL-20@GF	FS decreased from 108 N to about 252 N	^87^
CL-20@PDA/GO	FS decreased from 100 to 40%	^88^
CL-20@PEI/GO	FS decreased from 48 N to about 160 N	^89^
CL-20@M550/GO	FS decreased from 60 N to about 288 N	^90^
CL-20@551 glue/rGO	FS decreased from 100 to 28%	^91^
CL-20@NG	FS decreased from 72 N to about 108 N	^92^

## 5 Spheronization and Nanonization

According to the “hot spot” theory, spherical crystals have a smooth surface, which reduces the friction between solid particles compared to needle or flake crystals, reducing the probability of hot spot formation and thus reducing product sensitivity. The specific surface area is increased to spread the external force over more surfaces, and the force per unit area is greatly reduced as a result of nanosizing. When the specific surface area is larger, the surface energy of the particles is higher and the small particles can easily agglomerate. Under the action of an external force, the disintegration of the agglomerated particles will also consume part of the energy. In addition, the number of surface atoms of ultrafine explosives is large, making it easy to conduct heat conduction. When a hot spot is formed in an explosive, heat is transmitted from the inside of the explosive easily. All of the above factors show that spheronization and nanonization have a major influence on reducing its sensitivity.^94–96^

### 5.1 Ball Milling Method

The ball milling method is a common method to improve the sphericity of samples.^97^ The SEM image is shown in Figure 11a. It can be seen that the sample has no sharp edges and corners after ball milling, and its sphericity was close to 0.9, impact sensitivity decreased to 11.37 J, and the probability of friction explosion decreased to 40% (pendulum angle of 66°, sample mass of 20 mg, and pressure of 2.45 MPa). The particle size of CL-20 can be decreased significantly by the ball milling method.^98^ Compared to raw CL-20 (*D*_50_ = 15.96 μm), the impact and friction sensitivities of CL-20 after being milled (*D*_50_ = 200 nm) decrease significantly. The impact sensitivity and friction sensitivity of the sample after ball milling are 7.203 J and 66% (pendulum angle of 80 ± 1°, sample mass of 20 mg, and pressure of 2.45 MPa).

**Figure 11:**
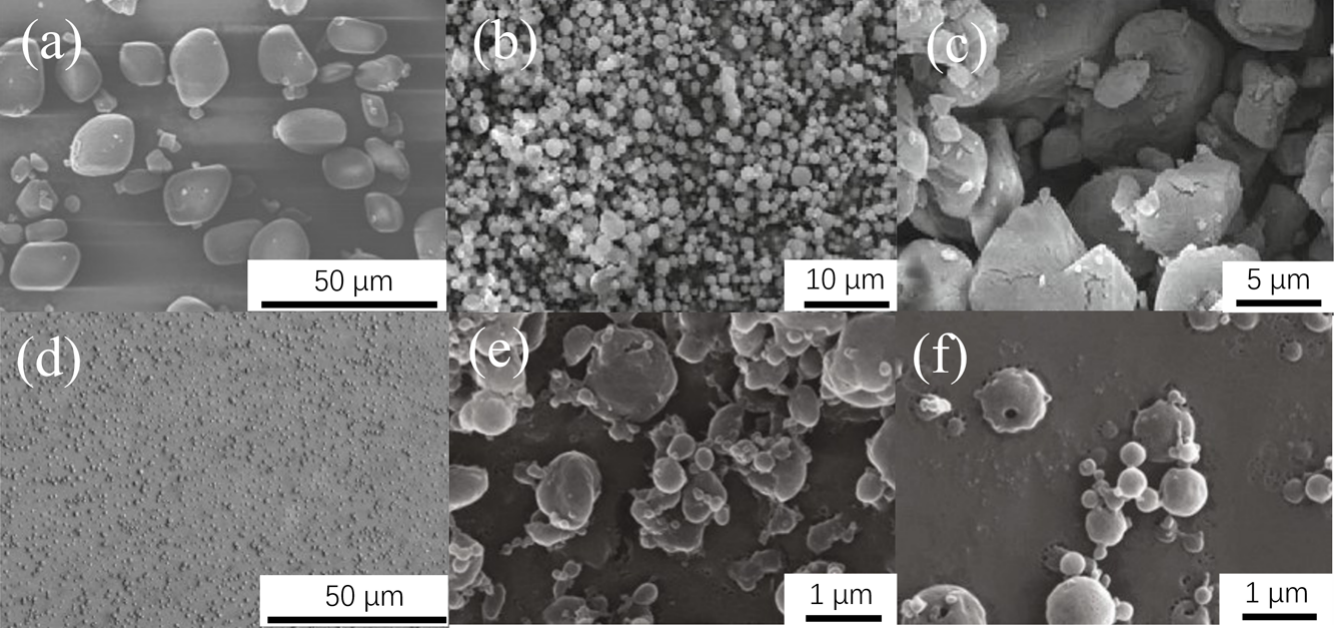
SEM images of CL-20 samples prepared by different methods: (a) ball milling method; (b) SDAS method; (c) spray recrystallization method; (d) ultrasonic-assisted spray method; (e) pressure ultrasonic-assisted spray refine method; and (f) siphon ultrasonic-assisted spray refine method. (Reproduced from refs 97,100,103,104 with permission.).

### 5.2 Spray Method

The spray technique can also be employed to fabricate spherical or nano CL-20.^99^ Furthermore, the spray-drying-assisted self-assembly (SDAS) method and spray recrystallization method have been applied to optimize CL-20, designated as CL-20–1 and CL-20–2, respectively.^100^ It can be seen from the SEM image (Figure 11b,c) that the sphericity of CL-20–1 is higher and the particle size is smaller compared to that of CL-20–2, which has a lower sensitivity. Sensitivity test results (Table 7) prove this point. For the spray crystallization method, CL-20 was dissolved in different solvents, sprayed, and collected after condensation.^101^ When ethyl acetate was used as the solvent and *n*-heptane was used as the nonsolvent, the crystal form of the obtained sample was the ε- crystal form and the particle size was 700∼900 nm. The test results show that the impact sensitivity decreases from 4.36 to 8.31 J.

**Table 7:** Test Results of Sensitivity Performance of Different Samples (Reproduced from Ref 100 with Permission)

sample	impact sensitivity/J	friction sensitivity/%
raw CL-20	3.23	100
CL-20–1	7.62	80
CL-20–2	5.54	92

### 5.3 Ultrasonic Method

The ultrasonic method facilitates enhanced dispersion of CL-20 particles and is frequently employed in conjunction with the spray technique.^102,103^ On the basis of the spray method, researchers have proposed the pressure ultrasonic-assisted spray refine method and the siphon ultrasonic-assisted spray refine method.^104^ The SEM image of the sample is shown in Figure 11e,f. It can be seen that the sample prepared by the siphon ultrasonic-assisted spray refine method has a higher sphericity. Compared with the pressure ultrasonic-assisted spray refinement method (7.47 J), the impact sensitivity of the sample (8.01 J) prepared by the siphon ultrasonic-assisted spray refinement method is also lower. The ultrasonic solvent–nonsolvent method is also used to prepare nano CL-20.^105^ The *D*_50_ of the sample after ultrasonic treatment is 95 nm, whose impact sensitivity is 10.78 J.

### 5.4 Summary

Spheronization and nanonization are other ways to reduce sensitivity. At present, research methods are mainly divided into three methods: ball milling method, spray method, and ultrasonic method.^106^ The ball milling method is convenient and cost-effective. The spray method means that CL-20 is dispersed in the solvent, and then the solution is sprayed out to form small droplets. After precipitation and crystallization, a fine powder can be obtained. In addition, the ultrasonic method is a further improvement on the spray method. After CL-20 is dispersed in the solvent, it is ultrasonically treated to make it disperse evenly and then sprayed. Compared with the spray method, the powder prepared by this method has a smaller particle size.

## 6 Analysis and Prospect

### 6.1 Crystal Control

Although researchers have done a lot of work on the crystal transformation mechanism of CL-20, so far the crystal transformation mechanism of CL-20 has not yet established a complete theoretical system. Most of the studies relate to the test and fitting of data (such as kinetic parameters). The influencing factors and causes of parameter changes have not been studied in depth. For the factors affecting the crystal structure, most studies only introduce the ultimate impact of a certain factor on the crystal structure without delving into the reaction process and principles in depth. In the future, the influence of different factors should be further studied, combined with developing advanced fabrication strategies, to prepare crystals with enhanced purity and minimized defect densities.

### 6.2 Cocrystallizations

The simulation research on cocrystals is relatively simple. Only the molecular molar ratios and crystal defects of some cocrystals have been studied for their impact on cocrystal sensitivity. In addition, almost all of the related simulations appear independently, and only a few researchers can combine simulations with experiments for more in-depth research.

For cocrystal preparation, a majority of the cocrystals are synthesized using only one method. Only a few types of cocrystals, such as CL-20/TNT, are prepared via different approaches. Therefore, it is necessary to explore the influence of different methods on the sensitivity of cocrystals and pay attention to the different effects caused by different process parameters. In addition, research scope on the selection of raw materials is still relatively narrow, and no studies have been found on the cocrystals of CL-20 and non-EMs. Currently, research on CL-20-based cocrystals is primarily concentrated on binary systems, while ternary cocrystal systems remain underexplored.^107^ Expanding studies to ternary systems could leverage multicomponent synergistic effects to achieve enhanced energy density, improved stability, and tunable sensitivity through diversified intermolecular interactions. This idea is in line with the urgent need for high-energy/low-sensitivity materials for advanced propulsion and explosive applications.

### 6.3 Surface Coating

For using EMs as coating materials, only some studies have explored the effect of component ratios on product sensitivity, while the influence of other factors such as preparation methods has not been discussed. With the development of carbon materials, options such as fluorinated graphene, oxidized graphene, and nitrated graphene have offered researchers more choices.^108–110^ However, current research mostly focused on the passivation effect of coating surfaces with different carbon materials without researching the specific process of coating with a certain carbon material or comparing the underlying reasons for the different passivation effects caused by different carbon materials. It is worth mentioning that although the selection of coating materials is becoming increasingly diverse, the safety and toxicity of surface coating materials must be considered.^111^

In addition, when the surface coating of CL-20 to reduce sensitivity is discussed, most explorations are conducted through experiments rather than simulations. Existing research mostly stands at the macro level, with limited attention given to micro issues such as the impact of interface binding energy on sensitivity. Common coating methods mainly include the spray method, the self-assembly method, and others. For the same group of materials, the influence of different methods was not considered. Due to the widespread application of surface coating in solving other chemical problems, researchers can refer to surface coating research in other fields rather than being limited to the field of EMs, which may help researchers consider how to reduce sensitivity through surface coating.

### 6.4 Spheronization and Nanonization

The ball milling method demonstrates notable advantages in terms of operational simplicity and economic feasibility. However, given the high sensitivity of CL-20, the ball milling method can significantly affect its stability, which makes the safety of this method relatively low. It is crucial to carefully adjust the process parameters during ball milling to prevent safety incidents. Nevertheless, there is still a lack of systematic research on summarizing the optimal ball milling process parameters. The properties of powders prepared by the spray method are closely related to process parameters such as the selection of solvent and also the nonsolvent. Nevertheless, few researchers have made a systematic exploration of this. Compared with the ball milling method, the advantage of the spray method, especially SDAS, is that it has higher safety and can produce powders with a smaller particle sizes and better sphericity, while the process is more complex and the cost is higher.

Thanks to simple processes and low costs, spheronization and nanonization are suitable for industrial production. However, the current relevant process parameters have not been systematically studied, which is a problem that needs to be solved.

### 6.5 Synergistic Effect

The sample data with a good desensitization effect in each part were integrated and analyzed. As illustrated in Figure 12, the desensitization effect progressively strengthens from spheronization and nanonization to crystal control, cocrystallizations, and surface coating. Compared with the general samples obtained by the solvent antisolvent method, the impact sensitivity significantly decreased. Among them, sphericity and nanocrystallization have the weakest desensitization effect, and the surface coating effect is the best, up to 35 J. Excitingly, these methods are not isolated. There are also a few studies that adopt multiple methods simultaneously to achieve the goal of reducing sensitivity, such as maintaining the ε- crystal form while surface coating,^112,113^ as well as preparing small-sized cocrystals^114–116^ and nanosized energetic composites,^117,118^ which is also an interesting and underexplored research direction. Over the years, such studies have witnessed a gradual upward trend. It is reasonable to anticipate that this synergistic effect will play a significant role in future desensitization research.

**Figure 12:**
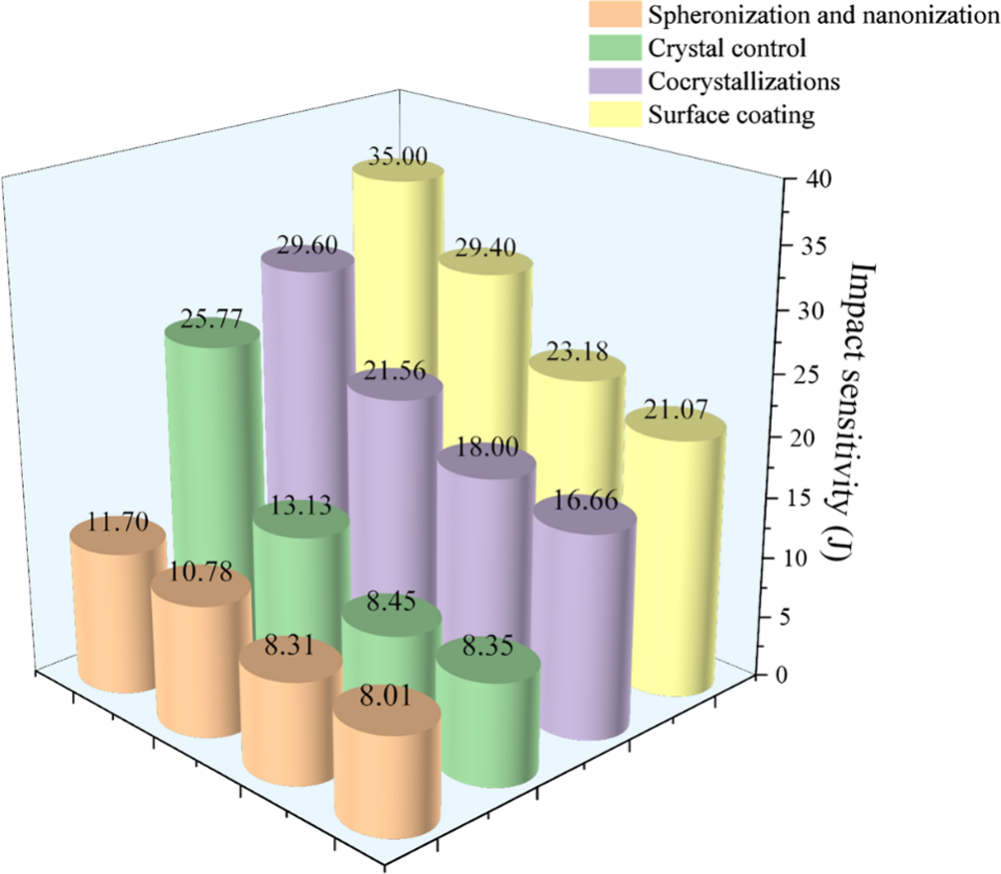
Analysis of samples with good desensitization effects prepared by four methods.

## 7 Conclusions

Various methods for reducing sensitivity to CL-20 are reviewed and discussed, mainly including crystal control, cocrystal coating, spheronization, and nanonization. Crystal control and cocrystals are both studied through experiments and simulations. The passivation effect, bonding mode, and cocrystal formation influencing factors between insensitive EMs and CL-20 are explored. Research indicates that MOFs have garnered significant research interest owing to their distinctive structural and functional properties. Meanwhile, carbon-based materials have become a promising material among many options. Notably, the CL-20@M550/GO composite demonstrates remarkably reduced impact sensitivity (35.00 J). Spheronization and nanonization are mainly divided into the ball milling method, spray method, and ultrasonic method. The process flow of the ball milling method is relatively simple, and particles with smaller particle size (95 nm) have been obtained by the ultrasonic method.

Despite the tremendous efforts made by researchers to reduce the sensitivity of CL-20, there are still many issues that urgently need to be addressed, for example, unclear crystal structure control principle, insufficient exploration of ternary crystals, ensuring that the energy level does not decrease significantly after coating to form a composite, and insufficient systematic research on the process parameters of spheronization and nanonization. In addition, the synergistic effect is like a mysterious treasure, waiting to be uncovered by researchers. All in all, CL-20 is a promising EM, and research on reducing sensitivity is key to its large-scale application.

## Glossary

ACM: acrylic rubber
AP: ammonium perchlorate
BTF: benzotrifuroxan
CaSt_2_: calcium stearate
CL-20/HNIW: 2,4,6,8,10,12-hexanitro-2,4,6,8,10,12-hexaazaisowurtzitane
DMF: N,N-dimethylformamide
DMSO: dimethyl sulfoxide
DNB: dinitrobenzene
DNDAP: 2,4-dinitro-2,4-diazapentane
DNI: dinitroimidazole
DNP: dinitrophenol
DOA: dioctyl adipate
FOX-7: 1,1-diamino-2,2-dinitroethylene
GAP: polyazide glycidyl ether
GF: graphene foam
GO: graphene oxide
HMX: octahydro-1,3,5,7-tetranitro-1,3,5,7-tetrazocine
HNB: hexanitrobenzene
LLM-105: 2,6-diamino-3,5-dinitropyrazine-1-oxide
M550: polyquaternium-7
MDNT: 1-methyl-3,5-dinitro-1,2,4-triazole
MOF: metal–organic framework
NC: nitrocellulose
NC_60_: nitrofullerene
NQ: nitroguanidine
NTO: 3-nitro-1,2,4-triazol-5-one
PBX: plastic bonded explosive
PDA: polydopamine
PEI: polyethylenimine
PETN: pentaerythritoltetranitrate
PNMAIW: 4,6,8,10,12-pentanitro-2-acetyl-2,4,6,8,10,12-hexaazaisowurtzitane
PW: paraffin wax
PXRD: powder X-ray diffraction
rGO: reduced graphene oxide
RDX: hexahydro-1,3,5-trinitro-1,3,5-triazine
SA: stearic acid
SCA2: 2-cyanoethyltriethoxysilane
TADNOIW: diacetyltetranitrohexaazaiso-wurtzitane
TAG: triaminoguanidine
TAGN: triaminoguanidine nitrate
TAGP: triaminoguanidine-glyoxal polymer
TATB: 1,3,5-triamino-2,4,6-trinitrobenzene
TFAZ: 7H-trifurazano [3,4-b:3′,4’-f:3”,4”-d]­azepine
TKX-50: dihydroxylammonium-5,5′-bistetrazole-1,1’-diolate
TNAD: trans-1,4,5,8-tetranitro-1,4,5,8-tetraazadecalin
TNB: trinitrobenzene
TNT: trinitrotoluene
TP: tea polyphenols
VitonA: copolymer of vinylidene fluoride and hexafluoropropylene
CED: cohesive energy density
EMs: energetic materials
DSC: differential scanning calorimetry
FTIR: Fourier transform infrared
TG: thermogravimetry analysis
SDAS: spray-drying-assisted self-assembly
SEM: scanning electron microscopy
SMPT: solution-mediated polymorphic transformation
XRD: X-ray diffraction
